# Comparing Hospital and Primary Care Physicians’ Attitudes and Knowledge Regarding Antibiotic Prescribing: A Survey within the Centre Region of Portugal

**DOI:** 10.3390/antibiotics10060629

**Published:** 2021-05-25

**Authors:** António Teixeira Rodrigues, João C. F. Nunes, Marta Estrela, Adolfo Figueiras, Fátima Roque, Maria Teresa Herdeiro

**Affiliations:** 1 Department of Medical Sciences, iBiMED—Institute of Biomedicine, University of Aveiro, 3800 Aveiro, Portugal; at.afonso@outlook.com (A.T.R.); mestrela@ua.pt (M.E.); teresaherdeiro@ua.pt (M.T.H.); 2 Centre for Health Evaluation and Research (CEFAR), National Association of Pharmacies, 1249 Lisbon, Portugal; 3 Department of Chemistry, CICECO—Aveiro Institute of Materials, University of Aveiro, 3800 Aveiro, Portugal; jcfn@ua.pt; 4 Department of Preventive Medicine and Public Health, University of Santiago de Compostela, 15702 Santiago de Compostela, Spain; adolfo.figueiras@usc.es; 5 Consortium for Biomedical Research in Epidemiology and Public Health (CIBER Epidemiology and Public Health—CIBERESP), 28001 Madrid, Spain; 6 Health Research Institute of Santiago de Compostela (IDIS), 15706 Santiago de Compostela, Spain; 7 Research Unit for Inland Development, Polytechnic of Guarda (IPG-UDI), 6300 Guarda, Portugal; 8 Health Sciences Research Centre, University of Beira Interior (CICS-UBI), 6200 Covilhã, Portugal

**Keywords:** hospital care, primary care, physicians, antibiotic, prescription, Portugal

## Abstract

Background: Antibiotic resistance is a worldwide public health problem, leading to longer hospital stays, raising medical costs and mortality levels. As physicians’ attitudes are key factors to antibiotic prescribing, this study sought to explore their differences between primary care and hospital settings. Methods: A survey was conducted between September 2011 and February 2012 in the center region of Portugal in the form of a questionnaire to compare hospital (*n* = 154) and primary care (*n* = 421) physicians’ attitudes and knowledge regarding antibiotic prescribing. Results: More than 70% of the attitudes were statistically different (*p* < 0.05) between hospital physicians (HPs) and primary care physicians (PCPs). When compared to PCPs, HPs showed higher agreement with antibiotic resistances being a public health problem and ascribed more importance to microbiological tests and to the influence of prescription on the development of resistances. On the other hand, PCPs tended to agree more regarding the negative impact of self-medication with antibiotics dispensed without medical prescription and the need for rapid diagnostic tests. Seven out of nine sources of knowledge’s usefulness were statistically different between both settings, with HPs considering most of the knowledge sources to be more useful than PCPs. Conclusions: Besides the efforts made to improve both antibiotic prescribing and use, there are differences in the opinions between physicians working in different settings that might impact the quality of antibiotic prescribing. In the future, these differences must be considered to develop more appropriate interventions.

## 1. Introduction

Antibiotic resistance is a worldwide public health problem, leading to longer hospital stays and raising medical costs and mortality levels [1,2,3,4,5]. Previous studies highlighted the role of the over-prescription and mis-prescription of antibiotics on the development of resistances [6,7,8,9,10,11]. Hence, promoting interventions to improve the antibiotic prescribing process is a key element to improving antibiotic use and diminishing resistances.

To improve the effectiveness of antimicrobial stewardship interventions, their design should be multifaceted, multidisciplinary and based on the characteristics of each specific setting [12,13,14]. However, prescribing is a complex process, usually affected by economic, demographic, clinical, cultural and social factors beyond evidence-based recommendations [6].

Attitudes are the key factors in the antibiotic prescribing process [13,15,16]. However, an in-depth understanding of how attitudes affect physicians’ clinical practice in different settings is needed. To our knowledge, there are no studies assessing the differences between attitudes and knowledge of hospital physicians (HPs) and primary care physicians (PCPs). Therefore, the aim of this study was to compare both the attitudes and knowledge between PCPs and HPs with regards to antibiotic prescribing.

## 2. Materials and Methods

### 2.1. Study Design, Population and Ethics Statement

A survey was conducted in Portugal’s Centre Regional Health Administration (ARS-Centro) (Population in 2011: ~1,737,059 people), from September 2011 to February 2012. Determinants of prescribing were assessed in all PCPs working in the National Health Service facilities of the ARS-Centro and hospital care physicians working in internal medicine departments within the hospitals of the ARS-Centro.

This study was approved by Portugal’s Centre Regional Health Administration (Permit No. 015650/2011), by the hospital’s administration and by the Portuguese Data Protection Authority (Comissão Nacional de Proteção de Dados/CNPD) (Permit No. 2886/2013). The personally addressed, reply-paid self-administered questionnaires were sent by post mail to PCPs up to four times to nonrespondents. In the case of HPs, the questionnaires were hand-delivered to the director of each hospital/medical service, who distributed them among the HPs. The answers were then collected by the administrative services of the department. The respondents did not receive any incentives. Nine hundred and fourteen questionnaires were delivered to PCPs and two hundred and twenty-seven questionnaires to HPs. All the data concerning PCPs’ knowledge and attitudes has been previously published [15].

### 2.2. Data Collection

All physicians were asked to fill a previously validated (published elsewhere [16,17]), two-page long questionnaire, divided into 5 sections:

1. Instructions to complete the questionnaire;
2. “Antibiotics and Resistance”: 17 statements regarding the knowledge and attitudes towards antibiotic prescribing, antibiotic use and antimicrobial resistance. To each of these statements, an attitude was attributed;
3. “In the treatment of respiratory infections, how would you rate the usefulness of each of these sources of knowledge?”: 9 statements regarding the importance of having several sources of knowledge, which can help comprehend the sources of knowledge underlying antibiotic mis-prescription;
4. Sociodemographic and professional data (age, gender, medical specialization, workplace and workflow);
5. Open box for additional comments.

The measurement of the agreement with the questions included in Section 2 and Section 3 of the questionnaire was performed through a horizontal, continuous visual analog scale, 8 cm long and unnumbered [16]. Answers were converted into a range from zero (total disagreement) to ten (total agreement). Physicians confidentiality was guaranteed.

### 2.3. Statistical Analysis

As the variables did not follow a normal distribution, nonparametric tests were conducted. Differences in the results between HPs and PCPs were evaluated using the Mann–Whitney *U* test. Differences were established as statistically significant at *p* < 0.05. The statistical analysis was performed using SPSS 25 (SPSS Inc., Armonk, NY, USA) and MS Excel (Microsoft Corporation, Redmond, WA, USA) software.

### 2.4. Sensitivity Analysis

In order to assess whether the differences observed were influenced by the fact that some physicians reported to work on both settings, a sensitivity analysis was conducted, in which these physicians were excluded.

## 3. Results

The overall response rate was 47.8%: of the PCPs; 421 answers were obtained, which corresponded to an overall response rate of 46.1%; 124 of the HPs invited accepted to participate in the study, corresponding to an overall response rate of 54.6%.

**Figure 1:**
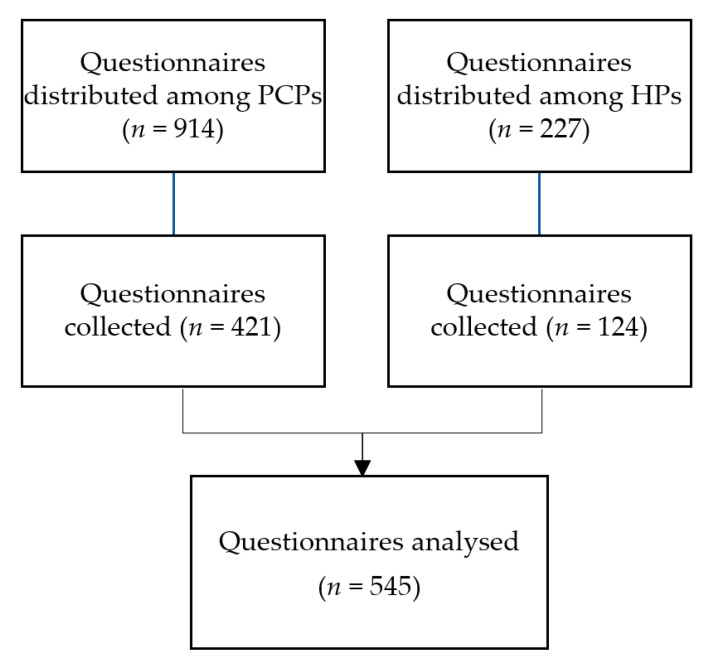
Questionnaire distribution and collection flowchart.

The process of distributing and collecting the questionnaires is summed up in the Figure 1 below.

### 3.1. Comparison of Sociodemographic and Professional Characteristics

Table 1 presents the comparisons between the sociodemographic characteristics of PCPs and HPs.

### 3.2. Comparison of Knowledge and Attitudes towards Antibiotic Prescribing, Antibiotic Use and Antimicrobial Resistance

Table 2 describes and compares the results obtained for the 17 statements concerning antibiotic prescribing and resistances in both PCPs and HPs.

Statistically significant results were obtained for the attitudes such as ignorance, responsibility of others, fear, complacency and indifference statements. Regarding ignorance, hospital physicians showed a higher agreement with resistance being a public health problem (S1), the importance of microbiological tests (S2) and the influence of prescriptions on the development of resistances (S4) compared to PCPs.

About the responsibility of others, HPs appear to be less convinced that the development of new antibiotics will solve the resistance problem (S5). On the other hand, PCPs tend to agree more regarding the negative impact of self-medication with antibiotics and dispense without medical prescription (S12 and S13). Furthermore, PCPs expressed a higher agreement about the need of rapid diagnostic tests (S3).

PCPs agreed more with the prescription of antibiotics in situations of fear and uncertainty (S7 and S8), as well as about having a complacent attitude with their patients (S10).

### 3.3. Comparison of the Usefulness of Different Sources of Knowledge

Table 3 describes and compares the results regarding the usefulness of different sources of knowledge between primary care and HPs.

The differences found in the usefulness of sources of knowledge were all related to HPs considering some of the sources evaluated more helpful. Differences were found for clinical practice guidelines, documentation from the industry and from medical information officers, continuous educations, contribution of specialists, peers and the internet.

### 3.4. Sensitivity Analysis

Overall, after the sensitivity analysis, the differences between PCPs and HPs, have remained constant in almost all dimensions. However, the attitudes of fear (S7) and responsibility of others—other professionals (S13) were no longer significant. Furthermore, despite that the distribution remained the same as the one reported in Table 2, the differences between the importance attributed to Continuing Education Courses (S6) were no longer significant.

## 4. Discussion

This study shows that, besides an overall increasing of both apprehension and knowledge regarding health professionals and patients, there are still differences in the knowledge and attitudes that may be important to tackle in future healthcare interventions [18,19]. Furthermore, and considering that antibiotics can only be prescribed by physicians, these results provide a picture on the discrepancies between HPs and PCPs in terms of attitudes and knowledge concerning antibiotic resistances.

Regarding the attitudes underlying antibiotic prescribing, statistically significant differences were found for the responsibility of others, fear, indifference, complacency and ignorance in 12 of the 17 statements evaluated. These constitute somewhat unsettling results, as it reveals how different the attitudes concerning resistance between both settings can be.

Most HPs and PCPs agreed that antibiotic resistance is an important public health problem in their settings, reflecting the knowledge regarding antibiotic resistance is a distressing worldwide public health problem, increasing medical costs and mortality levels [3,4,5,20,21]. However, significant differences were found between HPs and PCPs, where the HPs showed higher agreement with the statement. This fact might be related with the challenges that HPs are facing to hamper and control the spread of resistant infections and their treatment in their setting. HPs also showed higher knowledge when considering the impact of a prescription to a patient as a factor underlying the possible appearance of resistance. Prior evidence revealed antibiotic prescriptions and use as selective pressure driving at this resistance, both on an individual [22] and community level [23].

The development of new antibiotics is also a point of discordance between PCPs and HPs: the first are more convinced that new antibiotics will be developed, and the literature shows promising lines of research [24]. However, the evidence shows the importance of conserving the molecules already available in practice, namely by using them wisely.

Significantly different answers were also obtained regarding the usefulness of the microbiology results in deciding which treatment to provide and the availability of the diagnostic techniques. The results of blood cultures help to reduce antibiotic use and narrow antibiotic therapy, thus reducing the costs [25], and there are several emerging potential technologies that can address the clinical needs [26]. Nevertheless, the differences found might be related to the availability of diagnostic techniques in time in the hospital setting, contrarywise to what is found in primary care settings, in which widespread testing is not feasible. Yet, in some specific diseases—namely, for pharyngitis—rapid antigen testing is recommended before an antibiotic prescription [27,28,29,30,31].

Differences were also found regarding the responsibility of patients or pharmacies, where PCPs agreed more that patients may manage to obtain antibiotics even without a prescription and/or self-medicate. This difference in physicians’ perceptions might be related to the proximity between primary care and community pharmacy practices. Nevertheless, the evidence is published about the dispense without a prescription [32,33] and self-medication with antibiotics [34], which leads to antibiotic misuse and resistances.

Fear about future bacterial resistances was also an attitude where physicians showed differences. PCPs agreed more about prescribing broad-spectrum antibiotics, in the case of diagnostic uncertainty or the impossibility of following-up patients. This might be explained with the unavailability of diagnostic tools to aid physicians’ diagnosis in primary care settings and is concordant with the previous research [6,35,36,37].

The statistically significant opinions in which physicians showed the least agreement were related to insistence from the patient, patient trust and time constraints. Again, differences were found, and PCPs seem to be more complacent with patients than the HPs. There is data indicating that both physicians consider patient satisfaction is crucial, so it is vital to manage patients’ expectations [9,38], namely with emphasis on precise clarifications. As a patient’s satisfaction is related to their belief of their understanding of their disease, the data shows the success of longer, patient-centered consultations with low antibiotic prescriptions [6,8,9,13,39,40]. Changing primary healthcare setting is hard, but patient empowerment and reinforcement during doctor–patient communication with a realist awareness of the patient’s expectations was reveal to be essential in reducing the antibiotic prescription [41,42].

HPs tended to show higher agreement about the usefulness of the sources of knowledge. With exception to the courses promoted and organized by the pharmaceutical industry and previous clinical practices, all the other sources of knowledge evaluated were considered more useful by HPs. The reasons behind these differences are complex to understand, but the need to be continuously updated in a hospital care context might be associated with the results found. Furthermore, as the HPs tended to be younger, they might feel the need to resort to other sources of knowledge to compensate for a lower clinical practice experience when compared to PCPs, who tend to be older. Perhaps younger doctors rely more on sources of information based on scientific evidence, while older doctors rely on their clinical experience.

Considering the fact that the vast majority of antibiotics are prescribed in primary care [43], the identification of the differences in the attitudes and the underlying behaviors in antibiotic prescribing between settings allows us to tailor antibiotic stewardship interventions. As inadequate antibiotic prescriptions are an important factor for antibiotic resistances development [18,19], and high rates of inadequate antimicrobial prescribing are noted in the context of primary care [44], identifying the dimensions in which PCPs are less aware is an essential strategy toward improving these issues. Furthermore, parallel to the results in this study, in which PCPs tend to attribute more responsibility to others than HPs, and less important than the issue of antibiotic resistances, some studies have shown that PCPs do not only give less importance to the issue of antibiotic resistances but also tend to not consider themselves particularly accountable about their roles in this issue [20,21,44]. Hence, raising awareness among PCPs to tackle antimicrobial resistances should be a priority.

This study is accompanied by some limitations. Despite the statistically significant differences in attitudes, we cannot assure it triggers different antibiotic prescribing practices between settings. Considering the geographical limitations, the low response rate and specific characteristics regarding physicians’ clinical practice and the Portuguese Health System, the extrapolation of the results to other countries might not be adequate, as it can compromise its external validity. Nevertheless, the internal validity has been assured, as the questionnaire has been previously validated [16,45]. The response rates obtained both with primary care and HPs are low when compared to similar studies [46,47,48]. However, low response rates among physicians is a recognized problem in survey research [49,50]. The fact that the questionnaires were sent by post mail to PCPs and hand-delivered to the director of each hospital/service, along with the differences in the geographical dispersion of physicians within the territory, might explain the slight discrepancy between the response rates. Still, the difference in only 8% between response rates constitutes a positive outcome in this study, as it results in a less-biased appraisal between both settings. Furthermore, at the time in which this survey was conducted, there were 4.5 physicians/1000 inhabitants, considering both the public and private sectors [51]. Considering the estimated population of the center region, we estimate that this survey is highly representative of the public sector physician population, as it included almost a third of the physicians of this region. Even though this study was conducted in 2011 to 2012, which can be considered a limitation, the data from this study remains relevant, as the attitudes are stable variables over time [20,21], and it still brings an important contribution to the knowledge on this topic.

## 5. Conclusions

To conclude, the results of the study revealed that there are differences in opinions between physicians working in hospital and primary care that might impact the quality of antibiotic prescribing. Therefore, interventions to improve the antibiotic prescription quality should be tailored to each setting, especially considering the more evident differences between primary care and HP attitudes (particularly, dimensions of fear, ignorance and responsibility to others) for a more effective tackling of this global concern.

## Figures and Tables

**Table 1:** Sociodemographic and professional characteristics of both primary care and HPs.

	PCPs	HPs
**Age (years)**		
Median	55 (421/100%)	40 (124; 99.2%)
**Gender**		
Male	207/49.2%	44/35.5%
Female	214/50.8%	79/63.7%
Missing	0/0%	1/0.8%
**Activity in**		
Public practice	316/75.1%	99/79.8%
Public and private practice	95/22.6%	20/16.1%
Missing	10/2.4%	5/3.22%
**Setting**		
Primary care	309/73.4%	NA
Hospital and primary care	96/22.8%	25/20.2%
Hospital care	NA	98/79%
Missing	16/3.8%	1/0.8%
**Emergency Activity**		
No	133/31.6%	12/9.7%
Yes	280/66.5%	111/89.5%
Missing	8/1.9%	1/0.8%
**Patients per day** (25th and 75th percentile/*n*)	P25th = 20; P50th = 25; P75th = 30 *n* = 416/Missing = 5	P25th = 8; P50th = 10; P75th = 15 *n* = 124/Missing = 8
**Patients in emergencies per week** (25th, 50th and 75th percentile/*n*)	P25th = 15; P50th = 25; P75th = 40 *n* = 389/Missing = 32	P25th = 15; P50th = 20; P75th = 30 *n* = 124/Missing = 11
**Time (min) per** **consultation** (25th and 75th percentile/*n*)	P25th = 10; P50th = 15; P75th = 15 *n* = 385/Missing = 36	P25th = 20; P50th = 30; P75th = 30 *n* = 124/Missing = 13

**Table 2:** Differences in the attitudes and knowledge regarding antibiotic prescribing between PCPs and HPs (10—completely agree; 0—completely disagree).

	PCPs Percentiles			HPs Percentiles			*p*-Value
	25th	50th	75th	25th	50th	75th	
**S1:** Antibiotic resistance is an important Public Health problem in our setting (Ignorance).	8.5	9.5	10	9.5	10	10	<0.001
**S2:** In a primary care context, one should wait for the microbiology results before treating an infectious disease (Ignorance).	1	3.5	5.5	1.5	5	7	0.005
**S3:** Rapid and effective diagnostic techniques are required for diagnosis of infectious diseases (Responsibility of others—Health-care system).	6.5	9	10	4.5	7	9	<0.001
**S4:** The prescription of an antibiotic to a patient does not influence the possible appearance of resistance (Ignorance).	0.5	1.5	5	0.5	0.5	1.5	<0.001
**S5:** I am convinced that new antibiotics will be developed to solve the problem of resistance (Responsibility of others—Research)	3.5	5.5	8	3	5	6	<0.001
**S6:** The use of antibiotics on animals is an important cause of the appearance of new resistance to pathogenic agents in humans (Responsibility of others)	5.5	8	9.5	5	8	9.5	0.681
**S7:** In case of doubt, it is preferable to use a broad-spectrum antibiotic to ensure that the patient is cured of an infection (Fear).	2.5	5.5	8	1.5	4.5	7	0.045
**S8:** I frequently prescribe an antibiotic in situations in which it is impossible for me to conduct a systematic follow-up of the patient (Fear).	2	4	6	0.5	2.5	5	<0.001
**S9:** In situations of doubt as to whether a disease might be of bacterial aetiology, it is preferable to prescribe an antibiotic (Fear).	1	3	5.5	1	4	5.5	0.183
**S10:** I frequently prescribe antibiotics because patients insist on it (Complacency).	0.5	0.5	1.5	0.5	0.5	1	0.011
**S11:** I sometimes prescribe antibiotics so that patients continue to trust me (Complacency).	0.5	0.5	1	0.5	0.5	1	0.029
**S12:** I sometimes prescribe antibiotics, even when I know that they are not indicated because I do not have the time to explain to the patient the reason why they are not called for (Indifference).	0.5	0.5	1	0.5	0.5	1	0.009
**S13:** If a patient feels that he or she needs antibiotics, he or she will manage to obtain them at the pharmacy without a prescription, even when they have not been prescribed (Responsibility of others—Other Professionals).	4.5	6.5	9	3	5.5	8.5	0.026
**S14:** Two of the main causes of the appearance of antibiotic resistance are patient self-medication and antibiotic misuse (Responsibility of others—Patients).	8	9.5	10	6	8.5	10	0.011
**S15:** Dispensing antibiotics without a prescription should be more closely controlled (Responsibility of others—Health-care system).	9.5	10	10	9.5	10	10	0.613
**S16:** In a primary care context, amoxicillin is useful for treating most respiratory infections (Ignorance).	5.5	8.5	9.5	5.5	8.5	9.5	0.407
**S17:** The phenomenon of resistance to antibiotics is mainly a problem in hospital settings (Responsibility of others—Other professionals).	1	3	6	1	3.5	7.5	0.205

**Table 3:** Differences in the usefulness of sources of knowledge between primary care and HPs (10—completely agree; 0—completely disagree).

	PCPs Percentiles			HPs Percentiles			*p*-Value
	25th	50th	75th	25th	50th	75th	
**S1:** Clinical practice guidelines.	7	8.5	9.5	8	9	9.5	0.003
**S2:** Documentation furnished by the Pharmaceutical Industry.	2.5	5	5.5	3	5	6.5	0.019
**S3:** Courses held by the Pharmaceutical Industry.	2.5	5	6	2.5	5	7	0.223
**S4:** Information furnished by Medical Information Officers.	2	3.5	5.5	2	5	6	0.024
**S5:** Previous clinical experience.	7.5	8.5	9.5	7.5	8.5	9.5	0.408
**S6:** Continuing Education Courses.	7.5	8.5	9.5	8	9	9.5	0.019
**S7:** Others, e.g., contribution of specialists (microbiologists, infectious disease specialists, etc.).	7	8.5	9.5	8	9	9.5	0.005
**S8:** Contribution of peers (of the same specialisation).	6.5	8	9	7.5	9	9.5	<0.001
**S9:** Data collected via the Internet.	3	5.5	7.5	5	7	8.5	<0.001
